# Impact of amino acid substitutions in the V2 and C2 regions of human immunodeficiency virus type 1 CRF01_AE envelope glycoprotein gp120 on viral neutralization susceptibility to broadly neutralizing antibodies specific for the CD4 binding site

**DOI:** 10.1186/1742-4690-11-32

**Published:** 2014-04-23

**Authors:** Piraporn Utachee, Panasda Isarangkura-na-ayuthaya, Kenzo Tokunaga, Kazuyoshi Ikuta, Naokazu Takeda, Masanori Kameoka

**Affiliations:** 1 Thailand-Japan Research Collaboration Center on Emerging and Re-emerging Infections (RCC-ERI), Nonthaburi, Thailand; 2 National Institute of Health, Department of Medical Sciences, Ministry of Public Health, Nonthaburi, Thailand; 3 Department of Pathology, National Institute of Infectious Diseases, Tokyo, Japan; 4 Research Institute for Microbial Diseases, Osaka University, Osaka, Japan; 5 Department of International Health, Kobe University Graduate School of Health Sciences, Hyogo, Japan

**Keywords:** Human immunodeficiency virus type 1, CRF01_AE, Envelope glycoprotein, gp120, IgG1 b12, VRC01, Neutralizing antibody

## Abstract

**Background:**

The CD4 binding site (CD4bs) of envelope glycoprotein (Env) gp120 is a functionally conserved, important target of anti-human immunodeficiency virus type 1 (HIV-1) neutralizing antibodies. Two neutralizing human monoclonal antibodies, IgG1 b12 (b12) and VRC01, are broadly reactive neutralizing antibodies which recognize conformational epitopes that overlap the CD4bs of Env gp120; however, many CRF01_AE viruses are resistant to neutralization mediated by these antibodies. We examined the mechanism underlying the b12 resistance of the viruses using CRF01_AE Env (AE-Env)-recombinant viruses in this study.

**Results:**

Our results showed that an amino acid substitution at position 185 in the V2 region of gp120 played a crucial role in regulating the b12 susceptibility of AE-Env-recombinant viruses by cooperating with 2 previously reported potential N-linked glycosylation (PNLG) sites at positions 186 (N186) and 197 (N197) in the V2 and C2 regions of Env gp120. The amino acid residue at position 185 and 2 PNLG sites were responsible for the b12 resistance of 21 of 23 (>91%) AE-Env clones tested. Namely, the introduction of aspartic acid at position 185 (D185) conferred b12 susceptibility of 12 resistant AE-Env clones in the absence of N186 and/or N197, while the introduction of glycine at position 185 (G185) reduced the b12 susceptibility of 9 susceptible AE-Env clones in the absence of N186 and/or N197. In addition, these amino acid mutations altered the VRC01 susceptibility of many AE-Env clones.

**Conclusions:**

We propose that the V2 and C2 regions of AE-Env gp120 contain the major determinants of viral resistance to CD4bs antibodies. CRF01_AE is a major circulating recombinant form of HIV-1 prevalent in Southeast Asia. Our data may provide important information to understand the molecular mechanism regulating the neutralization susceptibility of CRF01_AE viruses to CD4bs antibodies.

## Background

The envelope glycoproteins (Env), gp120 and gp41, of human immunodeficiency virus type 1 (HIV-1) play a central role in viral transmission and mediate attachment and incorporation of the virus into target cells through specific interactions with the CD4 receptor and chemokine co-receptors [1]. In addition, Env is the major target of anti-HIV-1 neutralizing antibodies and, in particular, the CD4 binding site (CD4bs) of Env gp120 is a functionally conserved, important target of neutralizing antibodies [2-7].

Although numerous monoclonal antibodies against HIV-1 Env have been developed, a limited number of broadly reactive neutralizing human monoclonal antibodies (nhmAbs) have been established [8-11]. Two nhmAbs, IgG1 b12 (b12) and VRC01, are potent and broadly reactive neutralizing antibodies which recognize conformational epitopes that overlap the CD4bs of HIV-1 Env gp120 [5,7,12-14]. The nhmAb, b12 was established from a Fab (IgG1κ) phage display library generated from a bone marrow sample from an HIV-1-infected patient [12,15], and is able to neutralize diverse strains of HIV-1 [16,17]. In addition, b12 protects hu-PBL-SCID mice and macaque monkeys from infection with HIV-1 and a chimeric simian-human immunodeficiency virus (SHIV), respectively [18-20]. Moreover, adeno-associated virus vector-mediated gene transfer of the b12 gene protected humanized mice from HIV infection [21]. VRC01 is a potent and broadly reactive nhmAb established from an HIV-1-infected patient, and is capable of neutralizing diverse HIV-1 strains [5]. VRC01 inhibits HIV-1 infection in RAG-hu (SCID mice injected with human hematopoietic stem cells) mice [22] and hCD4/R5/cT1 (transgenic mice carrying the gene encoding human CD4, CCR5 and cyclin T1) mice [23]. It has been demonstrated that serum antibodies specific for the CD4bs of Env gp120 are responsible for the potent and broad neutralization of HIV-1 strains mediated by broadly reactive sera of HIV-1-infected patients [3]; therefore, it is important to establish a vaccine strategy to elicit broadly neutralizing antibodies against CD4bs, such as b12 and VRC01 [3,5,24]. To this end, the regulatory mechanisms underlying the susceptibilities of various HIV-1 strains to CD4bs antibodies, b12 and VRC01, need to be clarified.

CRF01_AE is a major circulating recombinant form (CRF) of HIV-1 prevalent throughout Southeast Asia [25]. In particular, CRF01_AE is responsible for more than 80% of infection cases in Thailand [26]. Although b12 is able to broadly neutralize HIV-1 subtypes B, C and D clinical isolates, it poorly neutralizes many CRF01_AE strains [5,16,27,28]. In addition, although VRC01 neutralizes 89% of CRF01_AE strains, the remaining 11% of the viruses are resistant to VRC01-mediated neutralization [5]. The mechanisms of how CRF01_AE viruses show low susceptibility or are resistant to neutralization by b12 and VRC01 are still not fully understood, and such studies are still ongoing [29-31]. Recently, we established a series of CRF01_AE Env (AE-Env)-recombinant viruses [32] and studied their neutralization susceptibility to nhmAbs including b12 [27,32]. Our study revealed that 2 potential N-linked glycosylation (PNLG) sites at amino acid positions 186 and 197, designated as N186 and N197 (amino acid numbering is based on the Env amino acid sequence of a reference strain, HXB2 [Genbank: K03455]), in the V2 and C2 regions of AE-Env gp120 play an important role in regulating the b12 susceptibility of AE-Env-recombinant viruses [33]. However, many AE-Env-recombinant viruses tested were still resistant to b12-mediated neutralization; therefore, we examined further the mechanism underlying the b12 resistance of AE-Env-recombinant viruses in this study.

## Results

### PNLG sites at amino acid positions 301, 339, 386 and 392 of Env gp120 play no major role in the b12 susceptibility of AE-Env-recombinant viruses

**Figure 1:**
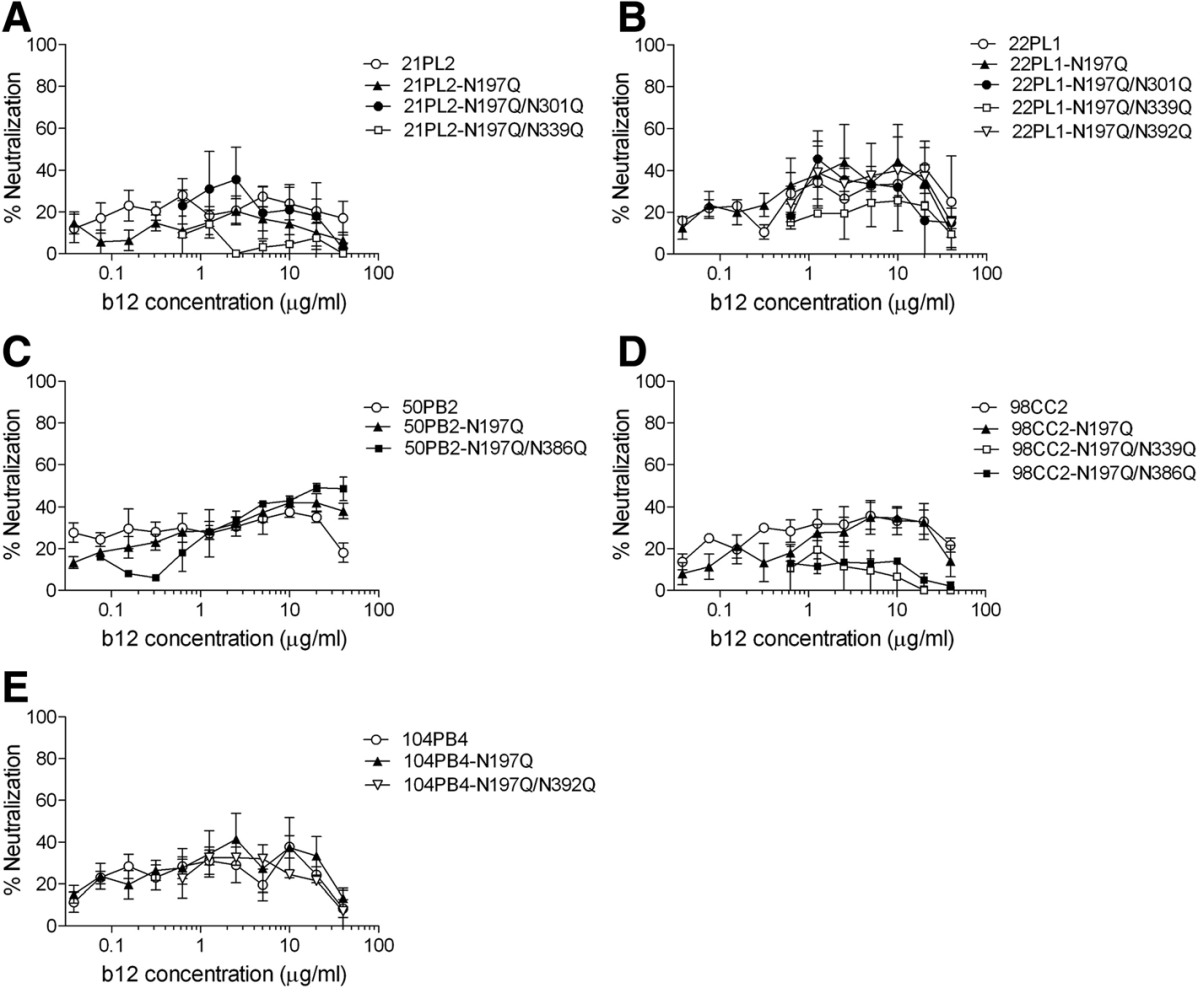
**The b12 susceptibility of N-linked glycan mutants of AE-Env clones.** Amino acid substitutions, N197Q, N301Q, N339Q, N386Q and N392Q were introduced into AE-Env clones, 21PL2 **(A)**, 22PL1 **(B)**, 50PB2 **(C)**, 98CC2 **(D)** and 104PB4 **(E)**. Then, the b12 susceptibility of recombinant viruses containing wild-type and mutant AE-Env clones was evaluated as described in Methods. The results are expressed as percent neutralization, which was calculated by determining the reduction in viral infectivity in the presence of b12 compared to that in control experiments in the absence of b12. All data points are the means and standard errors (error bars) of at least two independent experiments.

Our previous study showed the important role of 2 PNLG sites, N186 and N197, in the V2 and C2 regions of Env gp120 in regulating the b12 susceptibility of AE-Env-recombinant viruses [33]. Although most AE-Env-recombinant viruses tested were originally b12 resistant, the removal of N186 and/or N197 conferred b12 susceptibility to approximately 47% (15 of 32) of the recombinant viruses (Table 1). However, it was not possible to confer b12 susceptibility to the remaining 53% (17 of 32) of AE-Env-recombinant viruses (Table 1), indicating that other factors besides N186 and N197 are involved in the b12 resistance of AE-Env-recombinant viruses. Therefore, we searched for other determinants of the b12 susceptibility of AE-Env-recombinant viruses in this study. First, we compared the amino acid sequences involved in or in close proximity to the b12 contact sites of gp120, based on a previous report by Wu et al. [31], between 2 groups of AE-Env clones. One group consisted of 15 AE-Env clones which became b12 susceptible after removing N186 and/or N197, whereas the other group consisted of 17 AE-Env clones which were still resistant to b12 after removing these PNLG sites (Table 1). We found that 15 b12-susceptible AE-Env clones contained PNLG sites less frequently at amino acid positions 301 (N301), 339 (N339) and 392 (N392) of gp120 than 17 b12-resistant clones (Table 2). It was reported that N301 and a PNLG site at position 386 (N386) act as a glycan shield against neutralizing antibodies and may confer an advantage for transmission of CRF01_AE viruses from mother to infant [34]. In addition, N301, N339, N386 and N392 are involved in reducing the b12 susceptibility of subtype B Env (B-Env) [35-38]. Therefore, we examined the possible involvement of these PNLG sites in regulating the b12 susceptibility of AE-Env-recombinant viruses. To this end, a series of AE-Env mutants in which amino acid substitutions were introduced into these residues was prepared and tested their b12 susceptibility. The results showed that the introduction of amino acid substitutions into these PNLG sites did not improve the b12 susceptibility of selected AE-Env clones, 21PL2, 22PL1, 50PB2, 98CC2 and 104PB4 (Figure 1). In addition, the mutations, N339Q and N386Q, rather slightly reduced the b12 susceptibility of an AE-Env clone, 98CC2 (Figure 1D). Therefore, we concluded that these PNLG sites played no major role in regulating the b12 susceptibility of AE-Env clones.

**Table 1:** The b12 susceptibility of 32 AE-Env clones before and after removing N186 and/or N197

				**IC**_**50 **_**of b12 (μg/ml)**^**b**^		
	**PNLG site**^**a**^			**Mutation(s)**^**c**^		
**Env clone**^**a**^	**N186**	**N197**	**Wild-type**	**N186Q**	**N197Q**	**N186Q/N197Q**
29CC1	+ ^a^	+	>40^d^	>40	0.10	8.34
45PB1	+	+	>40	>40	>40	1.60
45CC1	+	+	>40	>40	0.02	0.65
47PL1	- ^a^	+	>40		0.03	
55PL1	-	+	>40		21.90	
62PL1	+	+	>40	>40	>40	0.77
65CC4	+	+	>40	1.31		
99PB2	-	+	>40		3.65	
99CC8	-	+	>40		0.10	
101PL1	+	+	>40	>40	>40	12.98
102CC2	-	+	>40		12.20	
105PB1	+	+	>40	>40	0.07	0.16
105PL2	-	+	>40		0.29	
105PL3	+	+	>40	>40	0.03	0.24
107CC2	+	+	>40	0.02		
21PL2	-	+	>40		>40	
22PL1	-	+	>40		>40	
41PB3	+	+	>40	>40	>40	>40
41CC1	+	+	>40	>40	>40	>40
47CC11	-	+	>40		>40	
50PB2	-	+	>40		>40	
50PL1	-	+	>40		>40	
52PB3	-	+	>40		>40	
52PL4	-	+	>40		>40	
52PL7	+	+	>40	>40	>40	>40
60PB2	-	+	>40		>40	
60PL2	-	+	>40		>40	
60CC3	+	+	>40	>40	>40	>40
98CC2	-	+	>40		>40	
98CC3	-	+	>40		>40	
99PL2	-	+	>40		>40	
104PB4	-	+	>40		>40	

^a^PNLG sites at the amino acid positions 186 (N186) and 197 (N197) were present (+) or absent (-) in the wild type of AE-Env clones. Amino acid numbering is based on the HXB2 Env gp120.

^b^IC_50_ of b12 for suppressing viral replication was calculated using GraphPad Prism 5 software. Data are shown as the means of at least two independent experiments.

^c^Single or multiple amino acid mutations were introduced into AE-Env-recombinant viruses.

^d^IC_50_ is >40 μg/ml.

**Table 2:** PNLG sites at positions 301, 339, 386 and 392 of gp120 in 32 AE-Env clones

		**PNLG sites**^**a**^		
**Env clone**^**b**^	**N301**	**N339**	**N386**	**N392**
HXB2	**NNT**	**NNT**	**NST**	**NST**
29CC1	NNV	YTV	**NTT**	**NNT**
45PB1	TNV	NKV	**NTT**	**NNT**
45CC1	KNV	NKV	**NTT**	**NNT**
47PL1	KHT	KQV	**NTT**	**NHT**
55PL1	**NNT**	SEV	**NTT**	T-T
62PL1	**NNT**	**NKT**	**NTT**	**NST**
65CC4	YET	FEV	**NTT**	DNT
99PB2	-NN	**NET**	**NTS**	**NIT**
99CC8	**NNT**	**NET**	**NTS**	**NIT**
101PL1	**NNT**	**NET**	**NTS**	SRT
102CC2	**NNT**	**NTT**	DTT	DS-
105PB1	**NNT**	NKV	**NTT**	**NKT**
105PL2	**NNT**	YEV	**NTT**	DNA
105PL3	**NNT**	NKV	**NTT**	**NKT**
107CC2	ESI	**NKT**	**NTT**	SST
21PL2	**NNT**	**NET**	**NTS**	**NTT**
22PL1	**NNT**	**NKT**	**NTT**	**NNT**
41PB3	**NNT**	YTV	**NTT**	NLN
41CC1	**NNT**	YTV	**NTT**	**NNT**
47CC11	**NNT**	KQV	**NTT**	**NHT**
50PB2	NNV	TEV	**NTT**	**NDT**
50PL1	**NNT**	VKV	**NTT**	**NNT**
52PB3	**NNT**	**NAT**	**NTT**	**NMT**
52PL4	**NNT**	**NAT**	**NTT**	**NMT**
52PL7	**NNT**	**NAT**	**NTT**	**NMT**
60PB2	**NNT**	**NQT**	**NTT**	**NQT**
60PL2	**NNT**	**NQT**	**NTT**	**NQT**
60CC3	GNR	**NQT**	**NTT**	**NQT**
98CC2	KNV	**NET**	**NTT**	**NNT**
98CC3	KNV	**NET**	**NTT**	**NNT**
99PL2	DNV	**NET**	**NTT**	PGR
104PB4	**NNT**	YKV	**NTS**	**NNT**

^a^The potential N-linked glycosylation (PNLG) sites at amino acid positions 301 (N301), 339 (N339), 386 (N386) and 392 (N392) were present or absent in the wild type of 32 AE-Env clones and HXB2. Amino acid sequences were aligned using the ClustalW algorithm with slight manual adjustment, followed by examining the potential N-linked glycosylation (PNLG) site using N-Glycosite (http://www.hiv.lanl.gov). PNLG sites are shown in bold text, while (-) represents a gap. Amino acid numbering is based on the HXB2 Env gp120.

^b^Fifteen AE-Env clones, 29CC1, 45PB1, 45CC1, 47PL1, 55PL1, 62PL1, 65CC4, 99PB2, 99CC8, 101PL1, 102CC2, 105PB1, 105PL2, 105PL3 and 107CC2 became b12 susceptible after removing PNLG sites, N186 and/or N197, whereas 17 AE-Env clones, 21PL2, 22PL1, 41PB3, 41CC1, 47CC11, 50PB2, 50PL1, 52PB3, 52PL4, 52PL7, 60PB2, 60PL2, 60CC3, 98CC2, 98CC3, 99PL2 and 104PB4, were b12 resistant after removing N186 and/or N197.

### A single amino acid substitution in the V2 region of gp120 significantly alters the b12 susceptibility of recombinant viruses containing AE-Env clones, 47CC11 and 47PL1

**Figure 2:**
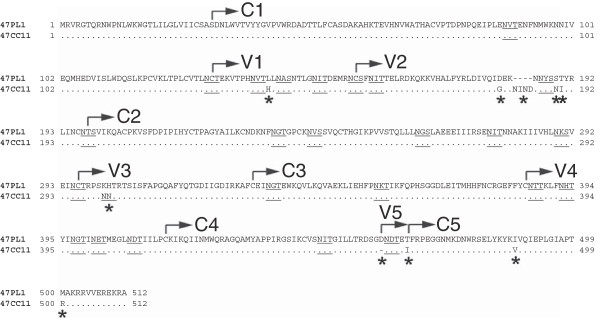
**Comparison of the amino acid sequences between 2 AE-Env clones, 47PL1 and 47CC11.** The deduced amino acid sequences of Env gp120 were compared between 2 AE-Env clones, 47PL1 [Genbank:EU743768] and 47CC11 [Genbank:EU743767]. The amino acid sequences were aligned using the ClustalW algorithm with slight manual adjustment. The positions of gp120 variable (V) and conserved (C) regions are denoted in the figure. Dots denote amino acid identity, while dashes represent gaps introduced to optimize alignment. PNLG sites are shown by underlining. The numbering of amino acid residues is based on the amino acid sequence of HXB2 Env and is shown beside the aligned sequences. Asterisks indicate the amino acid differences, a deletion, an insertion and an additional PNLG site found in the gp120 amino acid sequence of 47CC11 relative to that of 47PL1.

**Figure 3:**
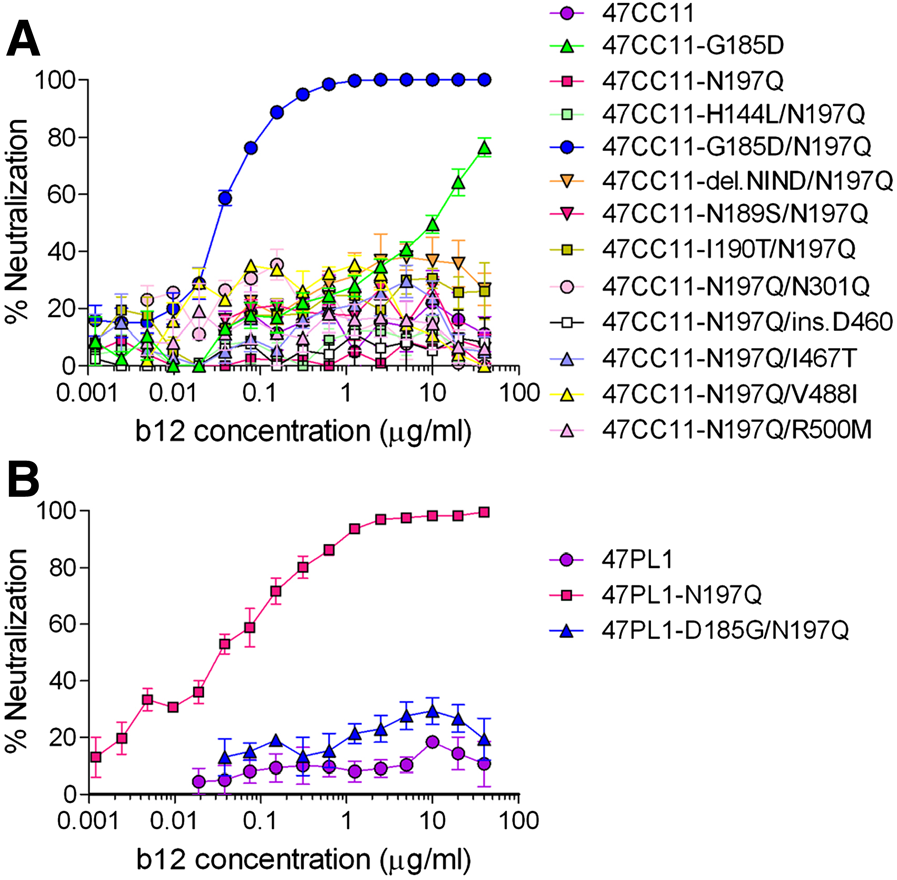
**The b12 susceptibility of wild-type and mutant AE-Env clones, 47CC11 and 47PL1.** The b12 susceptibility of recombinant viruses containing wild-type or mutant AE-Env clones, 47CC11 **(A)** and 47PL1 **(B)** was evaluated as described in Methods. The results are expressed as percent neutralization, as described in the legend to Figure 1. All data points are the means and standard errors (error bars) of at least three independent experiments.

Among 32 AE-Env clones, 2 AE-Env clones, 47PL1 and 47CC11, showed distinct neutralization susceptibility to b12 after the removal of N197 (Table 1), although these AE-Env clones were derived from an HIV-1-infected individual and showed a close phylogenetic relationship [32]. Namely, a recombinant virus containing 47PL1-N197Q (amino acid substitution from asparagine [N] to glutamine [Q] at position 197 in HXB2 numbering) was highly susceptible to b12 [50% inhibitory concentration (IC_50_) = 0.03 μg/ml], whereas the recombinant virus containing 47CC11-N197Q was resistant to b12-mediated neutralization (Table 1). In order to search for the determinants of b12 susceptibility, we compared the amino acid sequences of Env gp120 between 47PL1 and 47CC11. Ten positions were found to be different between b12-susceptible 47PL1 and b12-resistant 47CC11 (Figure 2, asterisks). Therefore, a series of point mutations, H144L, G185D, N189S, I190T, N301Q, I467T, V488I, R500M, del.NIND (deletion of 4 amino acid residues, NIND in the V2 region of gp120) (Figure 2) or ins.D460 (insertion of an amino acid residue D460) was introduced into 47CC11-N197Q, and recombinant viruses containing these AE-Env mutants were subjected to neutralization tests. The results showed that a recombinant virus containing 47CC11-G185D/N197Q became highly susceptible to b12 (IC_50_ = 0.03 μg/ml), whereas the 9 remaining 47CC11-N197Q-derived mutants were b12 resistant, similar to 47CC11-N197Q (Figure 3A). In addition, a mutant AE-Env clone, 47CC11-G185D, was constructed and subjected to neutralization tests. A recombinant virus containing 47CC11-G185D was moderately susceptible to b12-mediated neutralization (IC_50_ = 7.62 μg/ml), although the extent of b12 susceptibility was lower than that of 47CC11-G185D/N197Q (IC_50_ = 0.03 μg/ml) (Figure 3A). These results showed that an amino acid substitution, G185D, conferred b12 susceptibility to an AE-Env clone, 47CC11. In order to confirm the role of the amino acid substitution at position 185 in viral neutralization susceptibility to b12, a mutation, D185G, was introduced into 47PL1-N197Q, a b12-susceptible AE-Env mutant, and its effect on viral b12 susceptibility was tested. The result showed that a recombinant virus containing 47PL1-D185G/N197Q became b12 resistant, similar to the wild type of 47PL1 (Figure 3B). These results show that the amino acid residue at position 185 in the V2 region of gp120 plays an important role in determining the b12 susceptibility of 2 AE-Env clones, 47CC11 and 47PL1. Namely, aspartic acid (D185) and glycine (G185) residues at amino acid position 185 were responsible for the b12 susceptibility and resistance of 47PL1 and 47CC11, respectively.

### Comparison of amino acid residue at position 185 of Env gp120 among subtype B, subtype C and CRF01_AE viruses

We next studied the amino acid residue at position 185 of gp120 among HIV-1 subtype B, subtype C and CRF01_AE viruses, which were retrieved from the HIV sequence database. The results showed that 70.5% of B-Env clones, and 19% and 24% of subtype C Env (C-Env) and AE-Env clones, respectively, contained D185 (Table 3). In addition, a variation was observed in the amino acid residue at position 185 of gp120 among C-Env and AE-Env clones (Table 3). The majority of AE-Env clones contained D185 (24%), glutamic acid (E185) (27.5%), G185 (22%) or asparagine (N185) (16%) at position 185 of gp120 (Table 3). In addition, among our 32 AE-Env clones, D185 was conserved in 66.7% (10 of 15) of the clones which were b12 susceptible in the absence of N186 and/or N197, while G185, N185 or E185 was contained in 64.6% (11 of 17) of AE-Env clones which were resistant to b12 after removing N186 and/or N197 (Table 3).

**Table 3:** Amino acid residue at position 185 of HIV-1 Env

	**n**	**D185**^**a**^	**E185**^**a**^	**G185**^**a**^	**N185**^**a**^	**Others**^**a**^
Subtype B^b^	200	70.5%	6.5%	4.5%	7.5%	11%
Subtype C^b^	200	19%	15%	7.5%	36%	22.5%
CRF01_AE^b^	200	24%	27.5%	22%	16%	10.5%
b12 susceptible CRF01_AE^c^	15	66.7%	13.3%	6.7%	6.7%	6.7%
b12 resistant CRF01_AE^d^	17	11.8%	17.6%	17.6%	29.4%	23.5%

^a^Frequency (%) of samples with aspartic acid residue (D185), glutamic acid residue (E185), glycine residue (G185), asparagine residue (N185) or other residues, alanine, histidine, lysine, methionine, proline, glutamine, arginine, serine, threonine, valine or a gap (others) at amino acid position 185 of Env was estimated.

^b^200 *env* gene sequences of subtype B, C or CRF01_AE viruses with sampling dates 2008–2010, 2008–2009 or 2007–2010, respectively, were retrieved from the HIV sequence database (http://www.hiv.lanl.gov). The deduced amino acid sequences were translated and examined. Accession numbers are available upon request.

^c^AE-Env clones which became b12 susceptible after removing N186 and/or N197.

^d^AE-Env clones which were b12 resistant after removing N186 and/or N197.

### The impact of the amino acid residue at position 185 of gp120 on the b12 susceptibility of AE-Env-recombinant viruses

**Figure 4:**
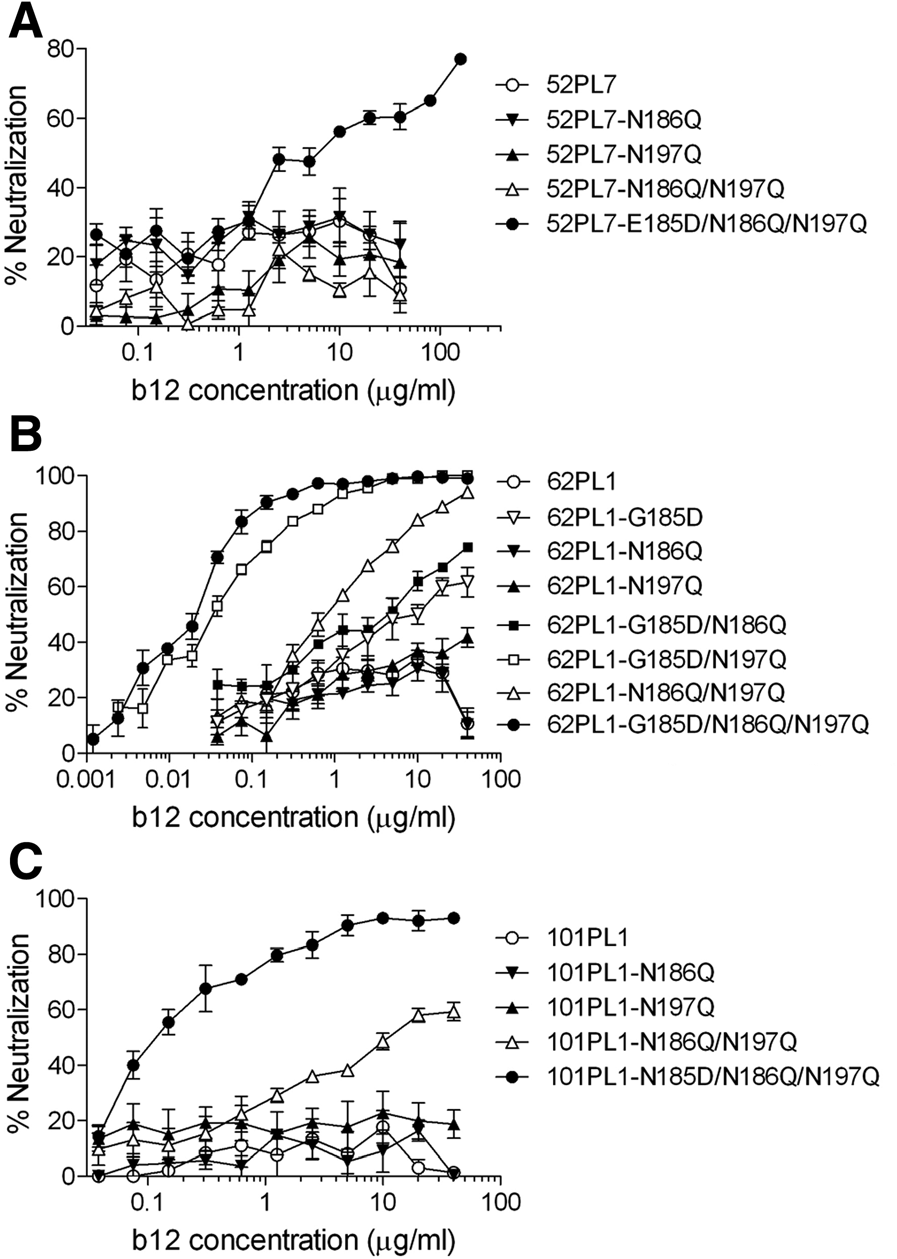
**The b12 susceptibility of wild-type and mutant AE-Env clones, 52PL7, 62PL1 and 101PL1.** The b12 susceptibility of recombinant viruses containing wild-type or mutant AE-Env clones, 52PL7 **(A)**, 62PL1 **(B)** and 101PL1 **(C)** was evaluated as described in Methods. The results are expressed as percent neutralization, as described in the legend to Figure 1. All data points are the means and standard errors (error bars) of at least three independent experiments.

We examined the role of the amino acid residue at position 185 in the b12 susceptibility of 3 selected AE-Env-recombinant viruses. The recombinant virus containing an AE-Env clone, 52PL7, was b12 resistant even after removing N186 and N197 (Figure 4A), while the recombinant viruses containing 62PL1 and 101PL1 became b12 susceptible at a low level after removing N186 and N197 (Figure 4B and C). However, the introduction of an amino acid substitution, E185D (Figure 4A), G185D (Figure 4B) or N185D (Figure 4C), conferred b12 susceptibility to AE-Env mutants or markedly improved their b12 susceptibility. Namely, the introduction of E185D conferred b12 susceptibility to an AE-Env clone, 52PL7-N186Q/N197Q (Figure 4A, 52PL7-E185D/N186Q/N197Q), while a mutation, G185D or N185D markedly improved the b12 susceptibility of 2 AE-Env clones, 62PL1-N186Q/N197Q (Figure 4B, 62PL1-G185D/N186Q/N197Q) and 101PL1-N186Q/N197Q (Figure 4C, 101PL1-N185D/N186Q/N197Q). In addition, although the single amino acid substitution, G185D, conferred b12 susceptibility to the wild type of 62PL1 (Figure 4B, 62PL1-G185D) (IC_50_ = 8.26 μg/ml), the extent of b12 susceptibility was further improved by multiple amino acid substitutions generating the mutants 62PL1-G185D/N186Q (IC_50_ = 2.32 μg/ml), 62PL1-G185D/N197Q (IC_50_ = 0.04 μg/ml) and 62PL1-G185D/N186Q/N197Q (IC_50_ = 0.01 μg/ml) (Figure 4B). These results suggested that 2 PNLG sites, N186 and N197, and the amino acid residue at position 185 synergistically regulated the b12 susceptibility of AE-Env clones.

We further examined the role of the amino acid residue at position 185, N186 and N197 on the b12 susceptibility of AE-Env clones using recombinant viruses, and the results are summarized in Tables 4 and 5. In addition, the relative infectivity of recombinant viruses containing wild-type or mutant AE-Env clones is shown in Tables 6 and 7. Most recombinant viruses containing mutant AE-Env clones maintained their infectivity, while some recombinant viruses lost their infectivity after the introduction of mutations (Tables 6 and 7). The wild types of 14 AE-Env clones, 21PL2, 47CC11, 50PB2, 52PB3, 52PL4, 52PL7, 60PB2, 60PL2, 62PL1, 65CC4, 98CC2, 101PL1, 102CC2 and 104PB4, were b12 resistant, and these AE-Env clones were still b12 resistant or showed comparably low levels of b12 susceptibility in the absence of N186 and/or N197; however, the introduction of an amino acid substitution, G185D, N185D or E185D, to these AE-Env mutants lacking N186 and/or N197, except 60PL2- and 104PB4-derived mutants, markedly improved their b12 susceptibility (Table 4). In addition, the removal of N186 and/or N197 conferred b12 susceptibility to 9 b12-resistant AE-Env clones, 29CC1, 45CC1, 47PL1, 99PB2, 99CC8, 105PB1, 105PL2, 105PL3 and 107CC2; however, the introduction of an amino acid substitution, D185G, to those AE-Env mutants lacking N186 and/or N197 transformed them into b12-resistant or low-susceptible mutants (Table 5). In addition, the introduction of a mutation, D185N or D185E, altered the b12 susceptibility of selected AE-Env clones, 29CC1, 45CC1, 47PL1, 105PB1 and 105PL3, to a lesser extent than the alteration by the introduction of D185G to the corresponding AE-Env clones (Table 5). These results suggested that D185 is responsible for the b12 susceptibility of AE-Env clones, while G185, N185 and E185 are responsible to a different extent for b12 resistance or the low susceptibility of AE-Env clones. Taking together the results shown in Figures 3 and 4 as well as in Tables 4 and 5, it is demonstrated that the amino acid residue at position 185 in the V2 region of gp120 plays a crucial role in the b12 resistance of AE-Env clones by cooperating with 2 PNLG sites, N186 and N197.

**Table 4:** The b12 susceptibility of AE-Env clones containing G185, N185 or E185, and the derived mutants

	**IC**_**50 **_**of b12 (μg/ml)**^**a**^												
	**Mutation(s)**^**b**^												
**Env clone**	**Wild-type**	**N186Q**	**N197Q**	**N186Q/N197Q**	**G185D**	**G185D/N186Q**	**E185D/N186Q**	**G185D/N197Q**	**N185D/N197Q**	**E185D/N197Q**	**G185D/N186Q/N197Q**	**N185D/N186Q/N197Q**	**E185D/N186Q/N197Q**
21PL2	>40^c^		>40						14.64				
47CC11	>40		>40		7.62			0.03					
50PB2	>40		>40						0.02				
52PB3	>40		>40							0.36			
52PL4	>40		>40							0.27			
52PL7	>40	>40	>40	>40									7.06
60PB2	>40		>40						16.19				
60PL2	>40		>40						>40				
62PL1	>40	>40	>40	0.77	8.26	2.32		0.04			0.01		
65CC4	>40	1.31					0.38						
98CC2	>40		>40		>40			5.07					
101PL1	>40	>40	>40	12.98								0.14	
102CC2	>40		12.20							0.23			
104PB4	>40		>40						>40				

^a^IC_50_ of b12 for suppressing viral replication was calculated using GraphPad Prism 5 software. Data are shown as the means of at least three independent experiments.

^b^Single or multiple amino acid mutations were introduced into AE-Env-recombinant viruses. Amino acid numbering is based on HXB2 Env gp120.

^c^IC_50_ is >40 μg/ml.

**Table 5:** The b12 susceptibility of AE-Env clones containing D185 and the derived mutants

	**IC**_**50 **_**of b12 (μg/ml)**^**a**^								
	**Mutation(s)**^**b**^								
**Env clone**	**Wild-type**	**N186Q**	**N197Q**	**N186Q/N197Q**	**D185G/N186Q**	**D185G/N197Q**	**D185N/N197Q**	**D185E/N197Q**	**D185G/N186Q/N197Q**
29CC1	>40^c^	>40	0.10	8.34		>40	12.59	>40	>40
45CC1	>40	>40	0.02	0.65		>40	5.77	0.45	>40
47PL1	>40		0.03			>40	>40	5.30	
99PB2	>40		3.65			>40			
99CC8	>40		0.10			>40			
105PB1	>40	>40	0.07	0.16		>40	7.03	>40	>40
105PL2	>40		0.29			>40			
105PL3	>40	>40	0.03	0.24		>40	10.09	>40	>40
107CC2	>40	0.02			3.42				

^a^IC_50_ of b12 for suppressing viral replication was calculated using GraphPad Prism 5 software. Data are shown as the means of at least three independent experiments.

^b^Single or multiple amino acid mutations were introduced into the AE Env-recombinant virus. Amino acid numbering is based on HXB2 Env gp120.

^c^IC_50_ is >40 μg/ml.

**Table 6:** The infectivity of AE-Env clones containing G185, N185 or E185, and the derived mutants

	**Relative infectivity (RLU)**^**a**^												
	**Mutation(s)**^**b**^												
**Env clone**	**Wild-type**	**N186Q**	**N197Q**	**N186Q/N197Q**	**G185D**	**G185D/N186Q**	**E185D/N186Q**	**G185D/N197Q**	**N185D/N197Q**	**E185D/N197Q**	**G185D/N186Q/N197Q**	**N185D/N186Q/N197Q**	**E185D/N186Q/N197Q**
21PL2	321		109						23				
47CC11	1080		670		617			927					
50PB2	283		296						356				
52PB3	1182		362							873			
52PL4	398		440							535			
52PL7	161	108	96	168									48
60PB2	366		404						93				
60PL2	748		996						908				
62PL1	297	447	495	300	696	745		533			78		
65CC4	88	29	0	0			105						
98CC2	437		103		579			104					
101PL1	130	149	50	33								152	
102CC2	1056		50							63			
104PB4	207		112						452				

^a^Infectivity of Env-recombinant virus was evaluated using U87.CD4.CXCR4 and U87.CD4.CCR5 cells. Relative infectivity of the virus was calculated by comparing it with the luciferase activity of pNL-envCT (pNL4-3)-infected U87.CD4.CXCR4 cells, which was defined as 100 relative light units (RLU).

^b^Single or multiple amino acid mutations were introduced into AE-Env-recombinant viruses. Amino acid numbering is based on HXB2 Env gp120.

**Table 7:** The infectivity of AE-Env clones containing D185 and the derived mutants

	**Relative infectivity (RLU)**^**a**^								
	**Mutation(s)**^**b**^								
**Env clone**	**Wild-type**	**N186Q**	**N197Q**	**N186Q/N197Q**	**D185G/N186Q**	**D185G/N197Q**	**D185N/N197Q**	**D185E/N197Q**	**D185G/N186Q/N197Q**
29CC1	573	587	773	704		708	371	364	662
45PB1	253	363	132	1					0
45CC1	464	780	302	357		243	167	194	68
47PL1	531		66			133	165	169	
99PB2	188		79			129			
99CC8	472		284			63			
105PB1	293	94	64	106		55	67	26	27
105PL2	581		640			397			
105PL3	355	128	146	39		108	83	31	26
107CC2	235	48	0	0	43				

^a^Infectivity of Env-recombinant virus was evaluated using U87.CD4.CXCR4 and U87.CD4.CCR5 cells. Relative infectivity of the virus was calculated by comparing it with the luciferase activity of pNL-envCT (pNL4-3)-infected U87.CD4.CXCR4 cells, which was defined as 100 relative light units (RLU).

^b^Single or multiple amino acid mutations were introduced into AE-Env-recombinant viruses. Amino acid numbering is based on HXB2 Env gp120.

### Correlation between the binding efficiency and neutralization susceptibility of AE-Env-recombinant viruses to b12

**Figure 5:**
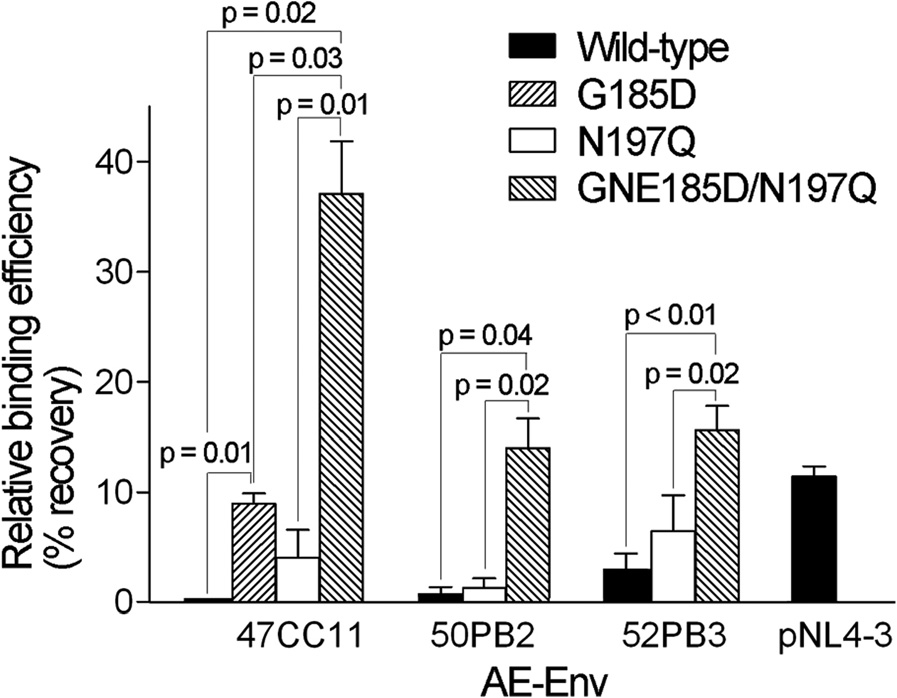
**The binding efficiency of b12 to Env proteins on viral particles.** Recombinant viruses containing wild-type AE-Env clones, 47CC11, 50PB2 and 52PB3, a wild-type B-Env clone, pNL4-3, and AE-Env mutants, 47CC11-G185D, 47CC11-N197Q, 47CC11-G185D/N197Q, 50PB2-N197Q, 50PB2-N185D/N197Q, 52PB3-N197Q and 52PB3-E185D/N197Q, were subjected to the study. The binding efficiency of b12 to viral particle-associated Env proteins was evaluated as described in Methods. Data are shown as the percent recovery of Gag p24 antigen, which was associated with virus-b12-magnetic bead complex relative to the input amount in an assay. The results are presented as the means and standard errors (error bars) of three independent experiments. Differences among the binding efficiencies of b12 to the wild-type and mutant AE-Env proteins were analyzed by the paired *t* test and are reported when *P* <0.05.

In our previous report, it was suggested that the N-linked glycosylation of amino acid residues at positions 186 and 197 of gp120 inhibited the binding of b12 to gp120 molecule in a computational model of the trimeric structure of HIV-1 Env proteins [33]. In order to study the mechanism of how a single amino acid substitution at the position 185 of gp120 regulated the b12 susceptibility of AE-Env clones, we tested the binding efficiency of b12 to AE-Env proteins on viral particles. The results showed that low or no binding of b12 to the wild-type Env proteins of three selected AE-Env clones, 47CC11, 50PB2 and 52PB3, was observed (Figure 5, closed bars). In contrast, the introduction of a single mutation, G185D or N197Q, somewhat improved the binding efficiency of b12 to these AE-Env proteins relative to the corresponding wild-type proteins (Figure 5, left hatched or open bars, respectively), while the introduction of double mutations, G185D, N185D or E185D (GNE185D), together with N197Q significantly improved the binding of b12 to AE-Env proteins (Figure 5, right hatched bars). A relative correlation was observed between the b12 binding efficiency (Figure 5) and the b12 susceptibility of AE-Env clones in each set of wild-type and mutant clones (Figure 3A and Table 4). These results suggested that the amino acid residue at position 185 regulated the b12 susceptibility of AE-Env clones at the level of the binding of b12 to Env proteins.

### The amino acid residue at position 185 of gp120 does not affect the b12 susceptibility of 5 B-Env-recombinant viruses, while the removal of a PNLG site, N186 or N197, improves the b12 susceptibility of 4 B-Env-recombinant viruses

**Figure 6:**
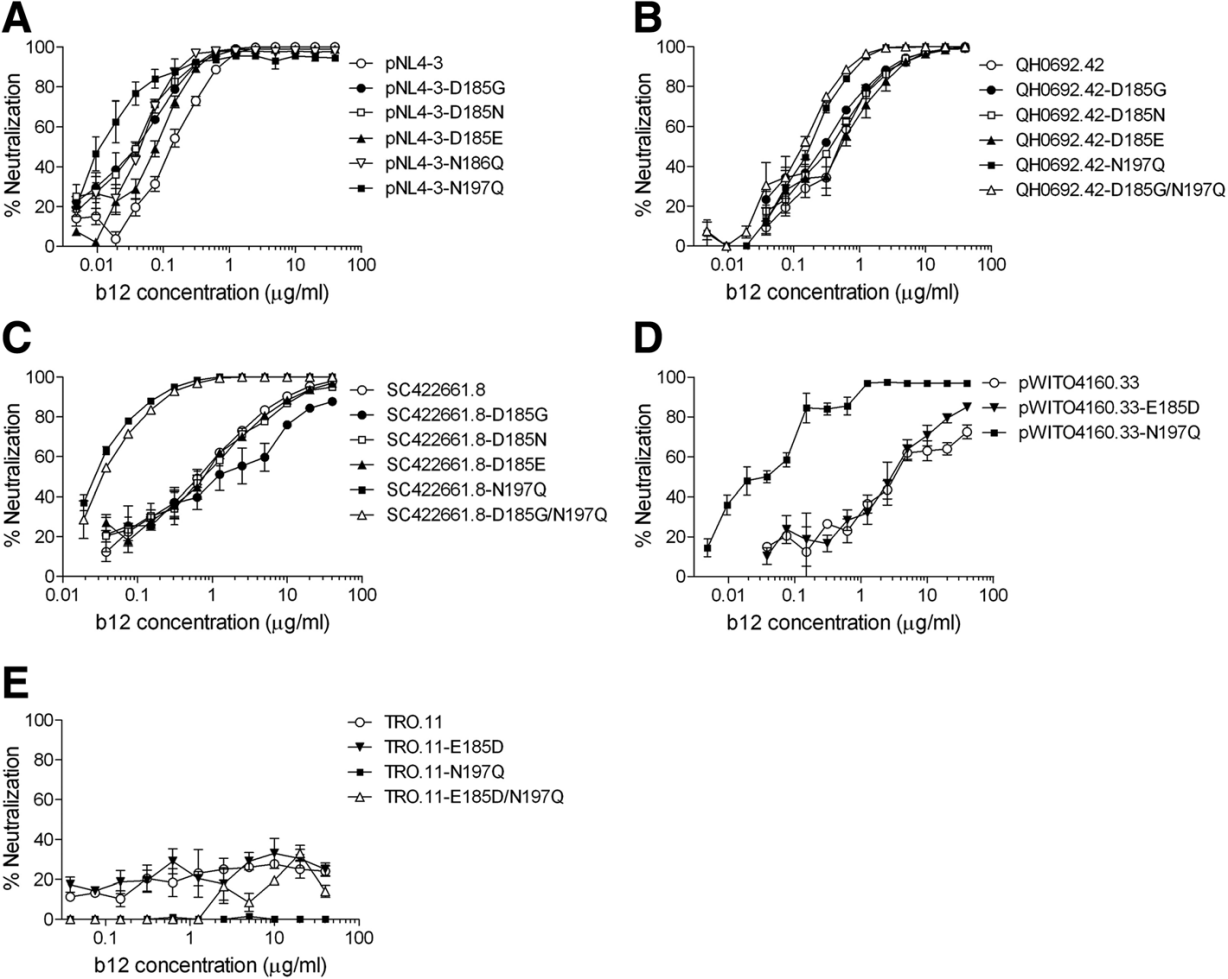
**The b12 susceptibility of wild-type and mutant B-Env clones.** The b12 susceptibility of recombinant viruses containing wild-type or mutant B-Env clones, pNL4-3 **(A)**, QH0692.42 **(B)**, SC422661.8 **(C)**, pWITO4160.33 **(D)** and TRO.11 **(E)** was evaluated as described in Methods. The results are expressed as percent neutralization, as described in the legend to Figure 1. All data points are the means and standard errors (error bars) of at least two independent experiments.

We next examined the role of amino acid residue at position 185 and 2 PNLG sites, N186 and N197, of gp120 in regulating the b12 susceptibility of 5 B-Env-recombinant viruses. Four recombinant viruses containing the wild-type Env of pNL4-3, QH0692.42, SC422661.8 and pWITO4160.33, were b12 susceptible, while a recombinant virus containing that of TRO.11 was b12 resistant (Figure 6). In addition, the wild-type Env of pNL4-3, QH0692.42 and SC422661.8 contained D185, while that of pWITO4160.33 and TRO.11 contained E185 (data not shown). Moreover, the wild-type Env of pNL4-3 contained N186 and N197, while that of QH0692.42, SC422661.8, pWITO4160.33 and TRO.11 contained N197, but not N186 (data not shown). We introduced an amino acid substitution at position 185; however, the b12 susceptibility of B-Env clones was not significantly altered by the introduction of the mutation (Figure 6). In contrast, the removal of N186 improved the b12 susceptibility of pNL4-3 (Figure 6A), while the removal of N197 improved the b12 susceptibility of pNL4-3, QH0692.42, SC422661.8 and pWITO4160.33 (Figure 6A, B, C and D), but not of TRO.11 (Figure 6E). In addition, the introduction of double mutations, E185D and N197Q, did not confer b12 susceptibility to TRO.11 (Figure 6E), while the introduction of double mutations, D185G and N197Q, did not alter the b12 susceptibility of QH0692.42 and SC422661.8, relative to the introduction of a single mutation, N197Q, into these B-Env clones (Figure 6B and C). Finally, mutant B-Env clones, pNL4-3-N186Q/N197Q, pNL4-3-D185G/N197Q and pWITO4160.33-E185D/N197Q were also constructed; however, recombinant viruses containing these B-Env mutants showed no infectivity (Table 8); therefore, we failed to test their b12 susceptibility. These results suggested that the amino acid residue at position 185 of gp120 played no major role in the b12 susceptibility of B-Env clones. In addition, 4 of 5 wild-type B-Env clones were already highly susceptible to b12 in the presence of N186 and/or N197 (Figure 6A, B, C and D), while the removal of N197 did not confer b12 susceptibility to TRO.11 (Figure 6E). Therefore, we consider that, although 2 PNLG sites, N186 and N197, of gp120 play an important role in regulating the b12 susceptibility of B-Env clones, the role for these B-Env clones is not as significant as that for AE-Env clones tested.

**Table 8:** The infectivity of B-Env clones and the derived mutants

	**Relative infectivity (RLU)**^**a**^									
	**Mutation(s)**^**b**^									
**Env clone**	**Wild-type**	**N186Q**	**N197Q**	**N186Q/N197Q**	**D185G**	**D185N**	**D185E**	**E185D**	**D185G/N197Q**	**E185D/N197Q**
pNL4-3	100	71	8	0	107	58	39		0	
QH0692.42	958		365		1187	809	870		377	
SC422661.8	657		324		569	19	503		395	
pWITO4160.33	49		5					109		0
TRO.11	294		69					306		107

^a^Infectivity of Env-recombinant virus was evaluated using U87.CD4.CXCR4 and U87.CD4.CCR5 cells. Relative infectivity of the virus was calculated by comparing it with the luciferase activity of pNL-envCT (pNL4-3)-infected U87.CD4.CXCR4 cells, which was defined as 100 relative light units (RLU).

^b^Single or multiple amino acid mutations were introduced into B-Env-recombinant viruses. Amino acid numbering is based on HXB2 Env gp120.

### Role of the amino acid residue at position 185, N186 and N197 in the VRC01 susceptibility of AE-Env-recombinant viruses

We next examined the role of the amino acid residue at position 185, as well as of 2 PNLG sites, N186 and N197, in the susceptibility of AE-Env-recombinant viruses to VRC01-mediated neutralization. VRC01 has been reported to be capable of neutralizing 89% of CRF01_AE viruses tested [5]; however, among selected AE-Env clones tested, the wild types of 56.5% (13 of 23) of AE-Env clones, 29CC1, 45CC1, 47PL1 and 105PL2 (Table 9) as well as 21PL2, 47CC11, 52PB3, 52PL4, 52PL7, 60PL2, 62PL1, 102CC2 and 104PB4 (Table 10), were VRC01 resistant, while those of 43.5% (10 of 23) of AE-Env clones, 99PB2, 99CC8, 105PB1, 105PL3 and 107CC2 (Table 9), as well as 50PB2, 60PB2, 65CC4, 98CC2 and 101PL1 (Table 10), were susceptible to VRC01-mediated neutralization. In addition, the VRC01 susceptibility of AE-Env clones, 45CC1, 99CC8, 105PL2, 105PL3 and 107CC2 (Table 9), as well as 21PL2, 50PB2, 52PB3, 52PL4, 60PB2, 60PL2, 65CC4, 98CC2, 101PL1 and 102CC2 (Table 10), was improved after the removal of N186 and/or N197, suggesting that 2 PNLG sites, N186 and N197, played a role in regulating the VRC01 susceptibility of some AE-Env clones tested. We next studied the role of the amino acid residue at position 185 in the VRC01 susceptibility of AE-Env clones. The results showed that the introduction of a mutation, D185G, to most AE-Env mutants lacking N186 or N197, except 45CC1- and 105PL2-derived mutants, did not reduce the VRC01 susceptibility of AE-Env clones (Table 9), suggesting that the amino acid residue at 185 had no major role in the VRC01 susceptibility of these AE-Env clones. However, the introduction of a mutation, G185D, N185D or E185D, to the AE-Env clones lacking N186 and/or N197 improved the VRC01 susceptibility of AE-Env clones, 21PL2, 50PB2, 52PB3, 52PL4, 60PB2 and 102CC2 (Table 10), while the introduction of the mutation did not affect significantly or rather reduced the VRC01 susceptibility of the remaining AE-Env clones, 47CC11, 52PL7, 60PL2, 62PL1, 65CC4, 98CC2, 101PL1 and 104PB4 (Table 10). These results suggested that the amino acid residue at position 185, as well as 2 PNLG sites, N186 and N197, regulated the susceptibility of some AE-Env clones to VRC01-mediated neutralization; however, their role in VRC01 susceptibility is not as significant as that for the b12 susceptibility of AE-Env clones.

**Table 9:** The VRC01 susceptibility of AE-Env clones containing D185 and the derived mutants

	**IC**_**50 **_**of VRC01 (μg/ml)**^**a**^				
	**Mutation(s)**^**b**^				
**Env clone**	**Wild-type**	**N186Q**	**N197Q**	**D185G/N186Q**	**D185G/N197Q**
29CC1	>2 ^c^		>2		>2
45CC1	>2		0.05		0.09
47PL1	>2		>2		>2
99PB2	0.02		0.10		0.03
99CC8	1.16		0.20		0.06
105PB1	0.07		0.11		0.04
105PL2	>2		0.07		0.09
105PL3	0.25		0.02		0.02
107CC2	0.33	0.15		0.09	

^a^IC_50_ of VRC01 for suppressing viral replication was calculated using GraphPad Prism 5 software. Data are shown as the means of at least two independent experiments.

^b^Single or multiple amino acid mutations were introduced into the AE Env-recombinant virus. Amino acid numbering is based on HXB2 Env gp120.

^c^IC_50_ is >2 μg/ml.

**Table 10:** The VRC01 susceptibility of AE-Env clones containing G185, N185 or E185, and the derived mutants

	**IC**_**50 **_**of VRC01 (μg/ml)**^**a**^										
	**Mutation(s)**^**b**^										
**Env clone**	**Wild-type**	**N186Q**	**N197Q**	**N186Q/N197Q**	**E185D/N186Q**	**G185D/N197Q**	**N185D/N197Q**	**E185D/N197Q**	**G185D/N186Q/N197Q**	**N185D/N186Q/N197Q**	**E185D/N186Q/N197Q**
21PL2	>2^c^		0.17				0.03				
47CC11	>2		>2			>2					
50PB2	0.07		0.03				0.01				
52PB3	>2		0.16					0.03			
52PL4	>2		0.25					0.08			
52PL7	>2			>2							>2
60PB2	0.28		0.08				0.04				
60PL2	>2		0.02				0.02				
62PL1	>2			>2					>2		
65CC4	1.20	0.28			0.32						
98CC2	0.38		0.19			0.21					
101PL1	0.50			0.04						0.12	
102CC2	>2		0.35					0.06			
104PB4	>2		>2				>2				

^a^IC_50_ of VRC01 for suppressing viral replication was calculated using GraphPad Prism 5 software. Data are shown as the means of at least two independent experiments.

^b^Single or multiple amino acid mutations were introduced into AE-Env-recombinant viruses. Amino acid numbering is based on HXB2 Env gp120.

^c^IC_50_ is >2 μg/ml.

## Discussion

Amino acid mutations and N-linked glycosylation of particular amino acid residues affect the protein structure and change the neutralization susceptibility of HIV-1 Env [39,40]. Although several reports describe the role of PNLG sites involved in or in close proximity to the b12 contact sites of gp120 in regulating the b12 susceptibility of subtype B viruses [31,34-38], the PNLG sites, N301, N339, N386 and N392, had no major role in the b12 susceptibility of AE-Env-recombinant viruses (Figure 1). We therefore searched for other determinants of the b12 resistance of AE-Env clones using recombinant viruses and found that a single amino acid substitution at position 185 in the V2 region of gp120 played a crucial role in regulating the b12 susceptibility of AE-Env clones by cooperating with two previously reported PNLG sites, N186 and N197, in the V2 and C2 regions of gp120 [33]. These amino acid positions were responsible for determining the b12 resistance of 21 of 23 (>91%) AE-Env clones tested. The V1/V2 regions of gp120 contact with CD4 molecule when gp120 binds to CD4 [41,42], and this may account for the role of V1/V2 regions in regulating viral susceptibility to neutralizing antibodies against the CD4bs of gp120, including b12 [35,43-46]. Previous computational analysis revealed that the amino acid residue at position 185 was involved in the regulation of viral b12 susceptibility [29]. The amino acid residue at position 185 locates near the C-terminus of V2 region, where the amino acid sequence is relatively conserved and affects the interaction of b12 with gp120 molecule [29,41,47]. In the report, G185 was suggested to be responsible for viral b12 resistance among several subtypes and CRFs of HIV-1, while D185, E185 or N185 of gp120 were responsible for viral b12 susceptibility [29]. Our results demonstrated the role of G185 in viral b12 resistance; however, they were partly inconsistent with the previous report. Namely, the effect of E185 and N185 on viral b12 susceptibility was not as significant as that of D185 in our study; therefore, there is a discrepancy between our biological and previous computational analyses.

Our previous report showed that high amino acid variability was observed in the V2 region, as well as in the V1, V4 and V5 regions, of Env gp120 among viral genomic fragments continuously collected for a short period (3 years) from CRF01_AE-infected Thai individuals [48], suggesting that the virus constantly evolved by introducing mutations in the V2 region of gp120, presumably in order to counteract anti-HIV-1 humoral immune responses. Env gp120 and gp41 are the major targets of anti-HIV-1 neutralizing antibodies, and are therefore candidates for vaccine antigens. Although an HIV vaccine has been under development for more than two decades, no effective vaccine is available [49]. Until recently, only one completed clinical trial in Thailand, RV144, was shown to have 31.2% protection efficacy against HIV-1 infection [49-51]. The recombinant Env gp120 protein derived from a CRF01_AE (A244) strain, A244-rgp120, and that derived from a subtype B (MN) strain, MN-rgp120, were used as immunogens in the RV144 clinical trial [52] and the majority of HIV-1 infection in the trial was caused by CRF01_AE viruses (91.7%) [53], which are the predominant CRF of HIV-1 prevalent in Thailand [26]. Recent analyses of the vaccine-induced immune responses in the RV144 trial showed that the induction level of antibodies against the V2 region of Env gp120 was inversely correlated with the infection risk [54-56]. In addition, antibodies against the V2 region recognized both a conformational epitope presented on a fusion protein containing the V1 and V2 regions of gp120, gp70-V1V2, as well as a linear epitope located at amino acid residues 165-178 in the V2 region [55], while a lysine residue at position 169 (K169) and an isoleucine residue at position 181 (I181) in the V2 region played an important role in determining vaccine efficacy [57]. These studies suggest that immunodominant regions located in the V2 region of Env gp120 are an effective target of protective immune response against CRF01_AE viruses, and the introduction of mutations into a few amino acid residues in the V2 region significantly affect the effectiveness of vaccine-induced anti-V2 neutralizing antibodies. Our study revealed that the major determinants of resistance to a CD4bs antibody, b12, were located in the V2 region of Env gp120 derived from CRF01_AE viruses. Therefore, we believe that further understanding of how amino acid mutations in the V2 region of Env gp120 affect the neutralization susceptibility of currently circulating CRF01_AE viruses to vaccine candidate-induced neutralizing antibodies as well as to established broadly reactive nhmAbs may provide important information to develop effective HIV-1 vaccines.

Our recent study showed that plasma samples derived from infected Thai individuals efficiently neutralize AE-Env-recombinant viruses, while the samples poorly neutralized B-Env- and C-Env-recombinant viruses [58], consistent with the results described in a previous report that serum samples derived from subtypes B and E (CRF01_AE)-infected Thai individuals showed subtype-specific neutralizing activity [59]. These results suggest a difference in the antigenicity of Env gp120 and gp41 among CRF01_AE, subtype B and C viruses. In addition, the AE-Env immunogen, A244-rgp120, is suggested to be able to induce a stronger antibody response against the V2 region than the B-Env immunogen, MN-rgp120, in the RV144 trial [55,60]; therefore, the immunogenicity of the V2 region of Env gp120 might also differ between CRF01_AE and subtype B viruses. Env gp120 is the most variable HIV-1 protein with typical intersubtype and intrasubtype differences soaring to 35% and 20%, respectively [61]. In addition, structural differences of the conserved and variable regions of Env gp120 are reported between subtype B and C Env molecules [62,63], while our previous study suggested that different Env regions are affected by host immune pressure between CRF01_AE and subtype B viruses [48]. Therefore, the structure of Env gp120 is somewhat different among diverse HIV-1 subtypes and CRFs. The b12 antibody recognizes a conformational epitope on gp120; thus, b12 susceptibility of the virus is necessarily affected by the protein structure of gp120. Our results showed that the amino acid residue at position 185 in the V2 region of Env gp120 played a major role in b12 susceptibility of AE-Env clones, but not of B-Env clones, suggesting that the structure of the V2 region may differ between CRF01_AE and subtype B viruses. Although the structure of the V2 region of Env gp120 derived from subtype C viruses is already determined [64], that of CRF01_AE has not been determined; therefore, we consider that it is important to solve the protein structure of AE-Env gp120 in order to design an effective vaccine antigen against CRF01_AE viruses. Since the structure of AE-Env gp120 including the V2 region is currently not available, it is difficult to discuss the structural aspects of the role of the amino acid residue at position 185 and 2 PNLG sites, N186 and N197, in regulating the b12 susceptibility of AE-Env clones; however, the potential effects of these amino acid substitutions on Env structure is as follows. According to the structural studies on BG505 SOSIP.664 gp140 derived from a subtype A strain, BG505, the glycan attached at the position 197 of gp120 contacts with the V3 region of neighboring gp120 molecule in Env trimeric structure, and is suggested to avoid the premature release of V3 region before the binding of gp120 to CD4 [65]. In addition, the removal of N197 from subtypes B and C Env proteins is reported to increase viral susceptibility to neutralizing antibodies including b12 [31,35,43,66,67], and this may be due to the changing in the quaternary structure of Env trimmers which leads to increase the accessibility of antibodies to the epitopes. The removal of N197 might reduce the stability of CD4-unbounded Env proteins and possibly decreases viral infectivity. However, the removal of N186 and N197, as well as the introduction of a mutation at amino acid position 185, did not significantly reduce the infectivity of most AE-Env-recombinant viruses (Tables 6 and 7), indicating that these AE-Env clones maintained the functional Env structure in the presence of these mutations. However, the binding of b12 to AE-Env proteins was significantly improved by the introduction of double mutations, G185D, N185D or E185D together with N197Q, but not by the introduction of a single mutation, N197Q or G185D (Figure 5). By considering these results, the introduction of an amino acid substitution at position 185 and the removal of N197 might synergistically alter the trimeric structure of AE-Env proteins and lead to increase the binding efficiency of b12 to Env proteins. We believe that our results might provide important information to take into account the antigenic and immunogenic diversity of Env gp120 among different subtypes and CRFs of HIV-1 to develop an effective HIV-1 vaccine.

## Conclusion

In this report, we show that the amino acid residue at position 185 and 2 PNLG sites in the V2 and C2 regions of AE-Env gp120 are the major determinants of viral resistance to CD4bs antibodies. We believe that our data may provide important information to understand the molecular mechanism regulating the neutralization susceptibility of HIV-1 CRF01_AE viruses to CD4bs antibodies as well as to design vaccine antigens against these viruses.

## Methods

### Cells

293T cells were maintained in Dulbecco’s modified Eagle’s medium supplemented with 10% fetal bovine serum (10% FBS-DMEM). U87.CD4.CCR5 and U87.CD4.CXCR4 cells were obtained from Drs. HongKui Deng and Dan R. Littman through the AIDS Research and Reference Reagent Program (ARRRP) (Division of AIDS, NIAID, NIH), and were maintained in 10% FBS-DMEM with puromycin (1 μg/ml) and G418 (300 μg/ml) (complete medium).

### Preparation of recombinant proviral constructs

cDNAs encoding full-length AE-Env gp120 and gp41 were cloned into pNL-envCT [68], a luciferase reporter proviral DNA derived from pNL4-3 [69], to generate AE-Env-recombinant proviral constructs as described previously [32]. In addition, full-length subtype B *env* clones, QH0692.42, TRO.11, pWITO4160.33 and SC422661.8 [70], obtained from Drs. Feng Gao, Beatrice H. Hahn, Ming Li, David C. Montefiori and Jesus F. Salazar-Gonzalez through the ARRRP, were cloned into pNL-envCT to generate B-Env-recombinant proviral constructs as described previously [58]. In order to generate N-linked glycosylation mutants, N186Q, N197Q, N301Q, N339Q, N386Q and/or N392Q, were introduced into proviral constructs by site-directed mutagenesis using the QuikChange site-directed mutagenesis kit (Agilent Technologies, Santa Clara, CA). In addition, single or multiple amino acid mutation(s), H144L, D185G, D185N, D185E, G185D, N185D, E185D, del.NIND (deletion of 4 amino acid residues, NIND), N189S, I190T, ins.D460 (insertion of D460), I467T, V488I and/or R500M, were introduced into proviral constructs by site-directed mutagenesis.

### Detection of PNLG site in the deduced amino acid sequence of HIV-1 *env* gene

PNLG sites in HIV-1 *env* genes were examined using N-Glycosite (http://www.hiv.lanl.gov).

### Preparation of recombinant virus

Viral supernatant was prepared by transfecting 293 T cells with a proviral construct using FuGENE HD transfection reagent (Roche, Basel, Switzerland). Forty-eight hours after transfection, the supernatant was cleared by centrifugation for 5 min at 8,000 rpm, and stored in aliquots at -85°C. The concentration of HIV-1 Gag p24 antigen in viral supernatants was measured by enzyme-linked immunosorbent assay (ELISA) (HIV-1 p24 Antigen Capture Assay; Advanced Bioscience Laboratory, Rockville, MD). The relative infectivity of recombinant viruses containing wild-type or mutant Env clones was examined, as follows. U87.CD4.CXCR4 or U87.CD4.CCR5 cells, which were seeded into a 24-well plate (3 × 10^4^ cells per 500 μl per well) 24 h prior to the tests, were infected with recombinant viruses (10 ng of p24 antigen). U87.CD4.CXCR4 cells were used as target cells for recombinant viruses containing CXCR4-tropic (X4) or dual-tropic (X4R5) Env, whereas U87.CD4.CCR5 cells were used as target cells for the viruses containing CCR5-tropic (R5) Env. Forty-eight hours after infection, luciferase activity in infected cells was measured using the Steady Glo Luciferase assay kit (Promega, Madison, WI) with an LB960 microplate luminometer (Berthold, Bad Wildbad, Germany). Relative infectivity of the recombinant virus was calculated by comparing it with the luciferase activity of pNL-envCT (pNL4-3)-infected U87.CD4.CXCR4 cells, which was defined as 100 relative light units (RLU).

### Neutralization tests

Neutralization susceptibilities of a recombinant virus to nhmAbs against the CD4bs of Env gp120, b12 (Polymun Scientific, Vienna, Austria) and VRC01 (obtained from Dr. John Mascola through the ARRRP), were examined as follows. Viral supernatants (5 ng of p24 antigen) were incubated with 2-fold serially diluted monoclonal antibody, b12 or VRC01, in 100 μl complete medium for 1 h at 37°C. U87.CD4.CXCR4 or U87.CD4.CCR5 cells, which were seeded into a 96-well plate (5 × 10^3^ cells per 100 μl per well) 24 h prior to neutralization tests, were then incubated with the mixture of viral supernatants and the antibody. Forty-eight hours after infection, luciferase activity in infected cells was measured as described above. Percent neutralization was calculated by determining the reduction in luciferase activity in the presence of the monoclonal antibody, b12 or VRC01, compared to that in control experiments in the absence of the antibody. The IC_50_ of the monoclonal antibody for suppressing viral replication was calculated using a standard function of GraphPad Prism 5 software (GraphPad Software, San Diego, CA).

### Binding assay

The binding efficiency of a recombinant virus to the monoclonal antibody, b12 was examined as follows. The antibody, b12 (5 μg) was incubated with protein G-conjugated magnetic beads, Dynabeads Protein G (0.3 mg) (Life Technologies, Lillestrom, Norway) with rotation for 24 h at 4°C in 400 μl phosphate-buffered saline (PBS), to generate b12-magnetic bead complex. After washed with PBS using the Dynal MPC-S Magnetic Particle Concentrator (Invitrogen Dynal, Oslo, Norway), the b12-magnetic bead complex was incubated with viral supernatants (2.5 ng of p24 antigen) with rotation for 24 h at 4°C in 100 μl of PBS. In a control experiment, viral supernatants containing an Env-deficient virus (2.5 ng of p24 antigen), that was generated from an *env*-deficient proviral construct, pNL-Luc-E^-^R^+^[71], was incubated with the b12-magnetic bead complex. Twenty-four hours after incubation, the virus-b12-magnetic bead complex was extensively washed with PBS and then HIV-1 Gag p24 antigen associated with the complex was measured by ELISA as described above. The percent recovery of Gag p24 antigen was calculated after subtracting the p24 antigen value associated with the virus-b12-magnetic bead complex in the control experiment using Env-deficient virus. Statistical analysis was carried out using a standard function of GraphPad Prism 5 software (GraphPad Software, San Diego, CA) with a Paired *t* test.

## Competing interests

The authors declare that they have no competing interests.

## Authors’ contributions

PU performed the majority of experiments, analyzed the data, designed the figures and drafted the manuscript. PI, KT, KI and NT contributed to design the study and participated in the analysis and interpretation of the data. MK designed the study and wrote the manuscript. All authors read and approved the manuscript.
